# A Case of Pott's Puffy Tumor in a Patient With Eosinophilic Chronic Rhinosinusitis

**DOI:** 10.7759/cureus.60893

**Published:** 2024-05-23

**Authors:** Shuya Tatsuki, Takeshi Tsuda, Kazuya Takeda, Sho Obata, Hidenori Inohara

**Affiliations:** 1 Otorhinolaryngology-Head and Neck Surgery, Osaka University Graduate School of Medicine, Suita, JPN

**Keywords:** ecrs, eosinophilic chronic rhinosinusitis, cranial surgery complications, frontal sinusitis, pott's puffy tumor

## Abstract

Eosinophilic chronic rhinosinusitis (ECRS) is a refractory type 2 inflammation-based airway allergic disease that is prone to complications such as bronchial asthma. Pott's puffy tumor (PPT) is a rare clinical entity characterized by osteomyelitis of the frontal bone accompanied by a subperiosteal abscess.

A 56-year-old female with a history of cranial surgery and bronchial asthma presented to an otolaryngology clinic with nasal obstruction and loss of smell, later developing swelling and redness on her forehead. She was diagnosed and treated for ECRS and was thought to have developed PPT during her course. Nasal endoscopy reveals bilateral polyp formation originating from the middle meatus. Paranasal computed tomography (CT) indicates substantial swelling extending from the opening of the frontal sinus to the adjacent subcutaneous tissue, accompanied by a defect in the frontal bone and osteolysis at the base of the frontal skull. Her management included conservative antibiotic therapy adjusted due to a drug eruption and, subsequently, endoscopic sinus surgery (ESS). The case was complicated by the patient's medical history and the absence of detailed surgical records, which limited the use of enhanced imaging techniques. This underscores the complexity of diagnosing and managing PPT in adults, particularly those with prior surgeries, emphasizing the need for a tailored diagnostic and therapeutic approach that integrates detailed patient history with current clinical indicators to effectively guide treatment. This case contributes to the limited literature on adult PPT and underscores the critical need for careful patient monitoring and detailed surgical history.

## Introduction

Pott's puffy tumor (PPT) is an uncommon yet severe complication of frontal sinusitis, initially described by Percivall Pott in the 18th century. This clinical entity is predominantly characterized by the development of frontal osteomyelitis accompanied by a subperiosteal abscess. These complications frequently precipitate further intracranial adversities such as epidural, subdural, and brain abscesses, particularly in patients with predisposing factors such as a history of trauma or prior cranial surgery [1,2].

Eosinophilic chronic rhinosinusitis (ECRS) is a distinct subtype of chronic rhinosinusitis, characterized by extensive nasal polyp formation and marked eosinophilic infiltration within the sinus tissue [3-5]. ECRS is recognized as a type 2 inflammatory airway disease involving various immune cells and molecules [6,7]. Current management strategies for ECRS involve a comprehensive approach that integrates surgical interventions with both local and systemic corticosteroid therapies. Despite these measures, patients often experience significant side effects and high recurrence rates, resulting in the inclusion of biologic therapies in treatment regimens for refractory cases.

This study aimed to delineate the diagnostic and therapeutic intricacies encountered in managing a patient presenting with PPT in the context of ECRS complicated by a previous surgical intervention in the cranial vault. This case underscores the critical importance of a detailed medical history and tailored therapeutic approach for managing complex sinonasal diseases.

## Case presentation

A 56-year-old female presented to an otolaryngology clinic in 2022 with primary complaints of loss of smell and nasal obstruction. The patient was initially managed conservatively as a suspected case of ECRS without surgical intervention. In 2023, she developed swelling and redness on her forehead, which prompted her to revisit the clinic, where she received antibiotic treatment. Despite this treatment, her symptoms worsened, prompting her referral to our department for comprehensive evaluation and further management.

**Figure 1:**
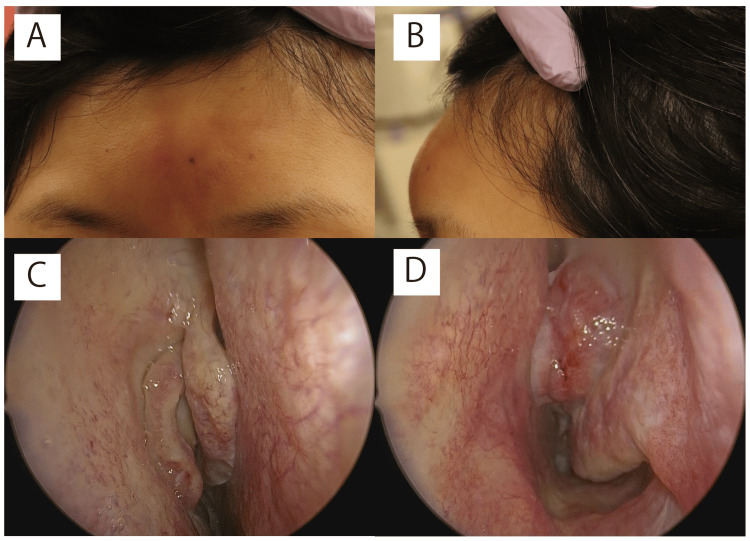
Initial physical examination A and B: Forehead redness and swelling observed. C and D: Polyps detected in both nasal cavities; no clear signs of purulent discharge.

At the initial visit, physical examination revealed clear consciousness, erythema, pain, and swelling of the frontal skin without neck rigidity (Figure 1A, 1B). Nasal sinus endoscopy revealed polyp formation in the bilateral middle meatuses (Figure 1C, 1D).

**Figure 2:**
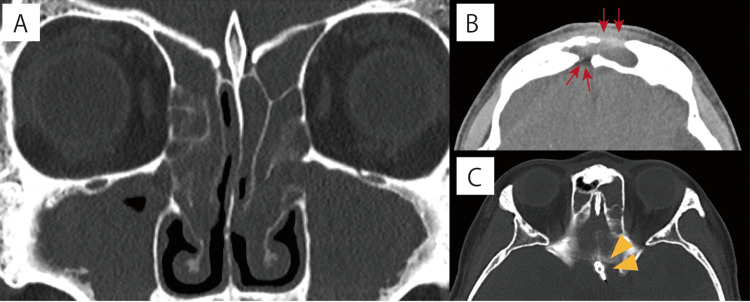
Preoperative CT scan A and B: There was a notable thickening of the subcutaneous tissue in the forehead; partial loss of the frontal bone and partial bony fusion at the posterior wall of the frontal sinus were observed (red arrows). C: Presence of a surgical clip (yellow triangles). CT: computed tomography

Blood tests indicated a white blood cell count of 7,510/μL, a neutrophil percentage of 67%, and a C-reactive protein level of 2.41 mg/dL. Her medical history included craniotomy, clipping surgery for a cerebral aneurysm approximately 30 years prior, and bronchial asthma. Contrast-enhanced computed tomography (CT) imaging was initially considered but was ultimately avoided due to complications from bronchial asthma. Magnetic resonance imaging (MRI) was similarly deferred because the details of the previous surgery were unclear and the clip material was unknown. Consequently, a plain paranasal CT scan revealed significant swelling from the opening to the surrounding subcutaneous area of the frontal sinus, with a frontal bone defect and osteolysis at the base of the frontal skull (Figure 2A-2C).

**Figure 3:**
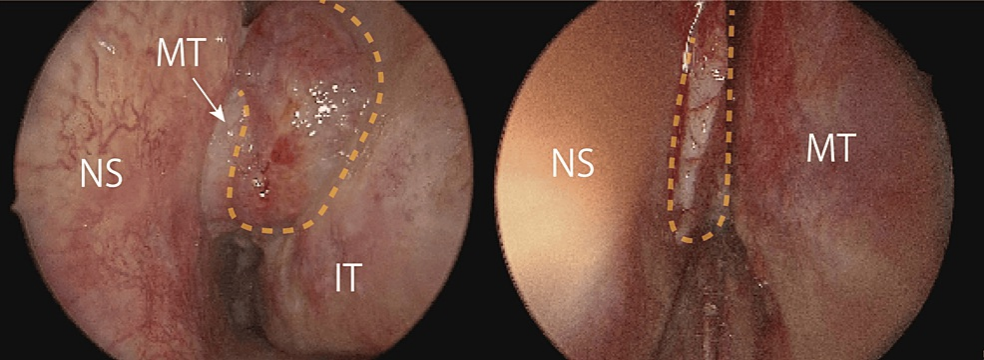
Intraoperative intranasal view Polyps observed in the middle meatus and olfactory cleft. The region demarcated by the yellow dashed line represents the nasal polyp. NS: nasal septum, MT: middle turbinate, IT: inferior turbinate

The initial diagnosis was suspected PPT due to an osteolytic lesion, contiguous extracranial abscess formation, and a history of cranial surgery. Intranasal findings and a history of bronchial asthma supported the co-diagnosis of ECRS. Ultrasound-guided incision and drainage of the forehead abscess were performed, and pus were collected for culture. Local anesthesia facilitated a 5 mm incision to drain the abscess, and gentamicin-applied gauze was used to prevent closure. Daily gauze changes and washing until the sixth day of hospitalization reduced the abscess discharge. With no evidence of intracranial infection, conservative antibiotic treatment was initiated, followed by surgery. The patient was administered 9 g/day of intravenous ampicillin/sulbactam (ABPC/SBT). The antibiotic dose was decreased to 6 g/day on the seventh day of inpatient treatment, following a reduction in swelling and pus drainage. However, on the ninth day after admission, the antibiotic was changed from ABPC/SBT to 4 g/day of ceftriaxone (CTRX) because of drug eruption throughout the body. Bilateral endoscopic sinus surgery (ESS) was performed 12 days after admission. Prior to surgery, the patient was treated with 10 mg of prednisolone (PSL) for seven days to minimize the nasal polyps and bleeding. During surgery, polyps were observed in the olfactory cleft and middle meatus (Figure 3).

**Figure 4:**
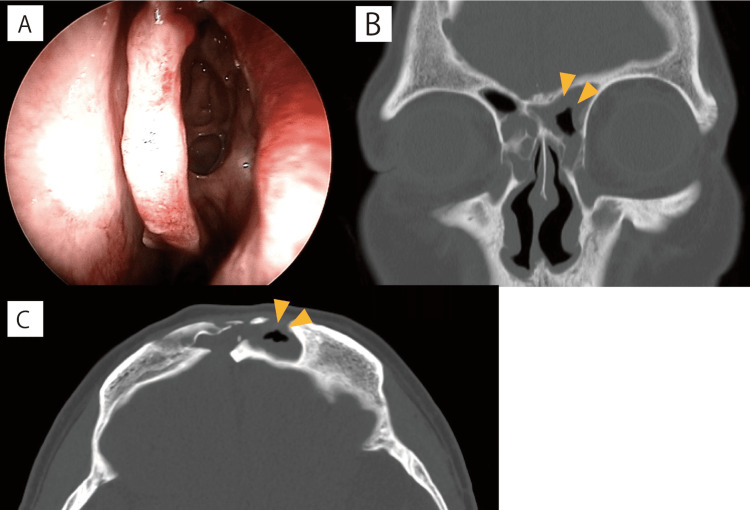
Four-month postoperative findings A: Intranasal observations. B and C: Mucosal thickening of the frontal sinus persists, although air entry is permitted (yellow triangles).

Drainage of pus from the maxillary sinus was observed, but there was no accumulation of pus around the nasofrontal duct. The polyps were removed, and the bilateral pan-sinus cavities were drained. Postoperatively, the patient continued PSL for six days and CTRX for seven days. Intranasal packing was removed on the 14th day, while nasal lavage was commenced soon after. The patient was discharged on the 19th day, transitioning to oral cephalexin (CEX) for 16 days. Four months post-discharge, while mucosal thickening persisted, air returned to the frontal sinus, warranting continued outpatient monitoring (Figure 4A-4C).

## Discussion

The clinical profile of PPT, as explored in this case, underscores its rarity and the complex etiology associated with previous cranial interventions. Historically, PPT has manifested primarily in the pediatric population, where it typically arises from the propagation of infection through the interosseous veins, resulting in frontal sinus osteomyelitis and subsequent subperiosteal abscess formation [8,9]. This pathophysiological mechanism contrasts significantly with those of the adult population, where PPT more commonly arises from prior trauma or surgical procedures, as exemplified in the current case. Notably, the patient's previous surgery, potentially involving nonbiodegradable materials such as bone wax, likely exacerbated local inflammatory responses, catalyzing the development of PPT. This correlation accentuates the peculiar vulnerability of postsurgical patients to such rare infections due to altered anatomical and vascular dynamics.

Furthermore, the standard diagnostic approach for PPT typically includes the use of imaging modalities such as CT and MRI to assess the extent of sinusitis and detect any intracranial complications [10,11]. In our case, a contrast-enhanced CT scan was difficult to perform because the patient had bronchial asthma. Additionally, the absence of detailed surgical records and the unknown composition of previously implanted clips significantly hindered the use of MRI. This limitation necessitated a reliance on clinical examination and non-contrast imaging to evaluate the patient's condition and plan for subsequent interventions. This situation starkly contrasts with typical cases where enhanced imaging facilitates a more comprehensive assessment and precise localization of abscesses, thus guiding therapeutic strategies more effectively.

Management of PPT often pivots on the severity of the associated intracranial complications. In the past, craniotomy was the preferred treatment for PPT; however, ESS has been increasingly favored for minimally invasive cases that present with no intracranial complications [12-14]. This was mirrored in our patient's treatment protocol, where initial conservative management with antibiotics and ultrasound-guided drainage was deemed sufficient due to the absence of neurological symptoms, followed by ESS rather than more invasive neurosurgical procedures. ESS is frequently chosen as a treatment option for ECRS. Endoscopic modified Lothrop procedure (EMLP) is sometimes selected, particularly in relapsed refractory cases, and was also considered in this case for manipulating the frontal sinus. However, EMLP carries the risk of postoperative bone thickening and stenosis. Considering the potential for causing a relapse of PPT due to bone thickening and the possibility of inadequate efficacy of antibody treatments, we opted for conventional ESS in this case. This decision reflected both the clinical presentation and the patient's complex medical history, emphasizing a personalized approach to managing PPT in the context of preexisting cranial surgery. This discussion delineates the critical need for a tailored diagnostic and therapeutic approach in managing PPT, especially in patients with significant medical histories.

## Conclusions

The absence of typical diagnostic tools due to safety concerns and the reliance on clinical judgment underscores the challenges faced in effectively treating complex cases while minimizing patient risks. This case not only highlights the nuances of managing PPT in adults with previous surgical interventions but also illustrates the importance of integrating patient history and current clinical indicators to guide treatment planning and execution.
